# Interleukin-8 Is Activated in Patients with Chronic Liver Diseases and Associated with Hepatic Macrophage Accumulation in Human Liver Fibrosis

**DOI:** 10.1371/journal.pone.0021381

**Published:** 2011-06-22

**Authors:** Henning W. Zimmermann, Sebastian Seidler, Nikolaus Gassler, Jacob Nattermann, Tom Luedde, Christian Trautwein, Frank Tacke

**Affiliations:** 1 Department of Medicine III, University Hospital, Aachen, Germany; 2 Institute of Pathology, University Hospital, Aachen, Germany; 3 Department of Medicine I, University of Bonn, Bonn, Germany; Singapore Institute for Clinical Sciences, Singapore

## Abstract

**Background:**

Interleukin-8 (IL-8, CXCL8) is a potent chemoattractant for neutrophils and contributes to acute liver inflammation. Much less is known about IL-8 in chronic liver diseases (CLD), but elevated levels were reported from alcoholic and hepatitis C-related CLD. We investigated the regulation of IL-8, its receptors CXCR1 and CXCR2 and possible IL-8 responding cells in CLD patients.

**Methodology:**

Serum IL-8 levels were measured in CLD patients (n = 200) and healthy controls (n = 141). Intrahepatic IL-8, CXCR1 and CXCR2 gene expression was quantified from liver samples (n = 41), alongside immunohistochemical neutrophil (MPO) and macrophage (CD68) stainings. CXCR1 and CXCR2 expression was analyzed on purified monocytes from patients (n = 111) and controls (n = 31). *In vitro* analyses explored IL-8 secretion by different leukocyte subsets.

**Principal Findings:**

IL-8 serum levels were significantly increased in CLD patients, especially in end-stage cirrhosis. Interestingly, patients with cholestatic diseases exhibited highest IL-8 serum concentrations. IL-8 correlated with liver function, inflammatory cytokines and non-invasive fibrosis markers. Intrahepatically, IL-8 and CXCR1 expression were strongly up-regulated. However, intrahepatic IL-8 could only be associated to neutrophil infiltration in patients with primary biliary cirrhosis (PBC). In non-cholestatic cirrhosis, increased IL-8 and CXCR1 levels were associated with hepatic macrophage accumulation. In line, CXCR1, but not CXCR2 or CXCR3, expression was increased on circulating monocytes from cirrhotic patients. Moreover, monocyte-derived macrophages from CLD patients, especially the non-classical CD16^+^ subtype, displayed enhanced IL-8 secretion *in vitro*.

**Conclusions:**

IL-8 is strongly activated in CLD, thus likely contributing to hepatic inflammation. Our study suggests a novel role of IL-8 for recruitment and activation of hepatic macrophages via CXCR1 in human liver cirrhosis.

## Introduction

Patients with chronic liver disease independent of its origin are prone to develop liver fibrosis and cirrhosis resulting in potentially lethal complications like esophageal varices or hepatic decompensation [1]. Inflammation is a key factor in the initiation and maintenance of fibrotic processes within the liver [2], [3]. During recent years, compelling evidence emerged that cellular components of the innate immune system such as monocytes and NK cells play major roles in perpetuating and modulating chronic liver inflammation [4], [5], [6]. Neutrophils, another important cell population of the innate immune response, have been intensively studied in acute liver damage such as alcoholic hepatitis [7], [8] or ischemia-reperfusion injury [9]. Increased endotoxemia, observed in patients with high ethanol intake, or ischemia-reperfusion lead to neutrophil accumulation in hepatic sinusoids and post-sinusoidal venules, resulting in extravasation and tissue infiltration. Furthermore, the degree of neutrophil infiltration in alcoholic hepatitis appears to be indicative of disease severity [10]. Yet, the contribution of neutrophils to chronic liver damage is less clear. For instance, it is controversial whether neutrophils are functionally intact or dysfunctional in patients with established cirrhosis [11], [12], [13], [14].

Apart from ‘classical’ inflammatory mediators like IL-1, TNFα and LPS, CXC-motif chemokines are critically involved in directing neutrophil trafficking towards sites of tissue injury [15]. IL-8 (CXCL8) is one important member of the CXC chemokine family with an additional characteristic glutamate-leucine-arginine (ELR) motif near the N-terminus of the molecule [16]. A key function of these ELR^+^ chemokines, such as CXCL8/IL-8, is the attraction of polymorphonuclear cells to sites of tissue injury and inflammation. Cytokines like IL-1 and TNFα are strong inducers of ELR^+^ chemokines [17]. IL-8 is synthesized by a vast number of different cells. Virtually all cell subsets possessing toll-like-receptors (TLR) are able to produce IL-8, among them monocytes and macrophages [18], [19]. CXCR1 and CXCR2 represent the cellular targets of IL-8 [20]. While CXCR1 binds almost exclusively IL-8, CXCR2 also shows high affinity to other CXC-chemokines (Gro-α/β/γ, NAP-2, ENA-78) [21]. Both, CXCR1 and CXCR2 can be found on neutrophils, monocytes/macrophages and other leukocyte subsets like T-cells [22], [23].

Several observations suggested that IL-8 pathways might be also involved in the pathogenesis of chronic liver disease. For instance, IL-8 levels are increased intrahepatically and in the serum of patients with alcoholic liver disease, probably contributing to hepatic neutrophil accumulation and also exerting systemic actions [24], [25]. In patients with chronic hepatitis C, IL-8 serum levels are associated with disease progression and relate to interferon unresponsiveness [26]. Interestingly, experimental work raised the possibility that IL-8 may not only act as a mere chemoattractant protein in the liver, but also exerts direct profibrogenic functions. As such, HCV core transduced Huh-7 cells were able to stimulate hepatic stellate cells (HSC) in an IL-8 dependent manner [27]. Likewise, IL-8 signalling has been linked to organ fibrosis in the prostate gland, pancreas and lung [28], [29], [30].

In this study, we investigated whether the IL-8 pathway is activated in patients with chronic liver disease (CLD). We demonstrate that IL-8 is upregulated in the circulation as well as in the liver of CLD patients, depending on disease severity and etiology. Activation of IL-8 is linked to progression of liver fibrosis in patients. Interestingly, monocytes/macrophages, and not neutrophils, appear to be main responders to IL-8 in liver fibrosis/cirrhosis via CXCR1. Moreover, the non-classical CD16^+^ monocyte subset is also a potent producer of this chemokine in humans.

## Materials and Methods

### Ethics Statement

Written informed consent was obtained from all participants. The study protocol conformed to the ethical guidelines of the 1975 Declaration of Helsinki as reflected in *a priori* approval by the local ethics committee of the University Hospital Aachen. The ethics committee of the University Hospital Aachen, RWTH-University, Aachen, Germany, specifically approved this study.

### Patients and controls

We investigated CLD patients with or without liver cirrhosis [4]. The presence of liver cirrhosis was assessed according to various imaging techniques (liver ultrasound, CT- or MRI-scan), biopsy or laparoscopy findings and if typical complications of liver cirrhosis (e.g. esophageal varices, ascites, hepatic encephalopathy) were present in conjunction with the history of chronic liver disease (Table 1). As controls, healthy volunteers with normal aminotransferases and negative serology for HBV, HCV and HIV were recruited from the local blood transfusion department.

**Table 1:** Characteristics of the study population.

	all patients	Stage of cirrhosis			
		no cirrh.	Child A	Child B	Child C
n	200	75	45	37	43
sex male/female n	124/76	46/29	24/21	20/17	34/9
age median (range) yrs	53 (17–82)	43 (17–73)	62 (30–82)	59.5 (28–77)	53 (21–81)
disease etiology n					
virus hepatitis	75	43	18	10	4
biliary/autoimmune	27	14	7	4	2
alcohol	60	5	13	14	28
other origin	38	13	7	9	9
MELD score median (range)	n.a.	n.a.	8 (6–17)	12 (7–21)	17 (9–28)
ALT median (range) U/L	46 (7–2103)	68 (10–2103)	41 (8–1247)	40.5 (7–437)	46 (11–261)
bilirubin median (range) mg/dl	1.3 (0.2–43.2)	0.7 (0.3–23.9)	0.9 (0.2–8.9)	2.05 (0.4–12.2)	4.7 (0.6–43.2)
albumin median (range) mg/dl	38 (12–61)	44.9 (29–61)	39 (28–49)	30.7 (12–43)	26 (12–60)
hyaluronic acid median (range) µg/l	190 (11–800)	31 (10–530)	240 (28–800)	500 (140–800)	740 (55–800)
procollagen-III-peptide median (range) µg/l	1160 (414–10400)	910 (414–1750)	1010 (456–3140)	1960 (639–10400)	2120 (696–6560)
IL-8 median (range) pg/ml	14 (14–1986)	15 (14–588)	28 (14–2530)	53 (14–1533)	88 (14–1533)

n.a., not applicable.

In a subgroup of patients, liver tissue samples were available from routine liver biopsies. Cirrhotic tissue was obtained from explanted livers due to transplantation. Grading and staging of all liver specimens was performed according to Desmet-Scheuer score by a pathologist who was blinded for experimental data [4]. Normal liver samples were obtained from unaffected areas of liver resections for secondary liver malignancy (mostly metastasis of colorectal cancer).

### IL-8 protein measurements

IL-8 protein in serum and cell culture supernatant was assessed using the FlowCytomix System (eBioscience) according to manufacturer's instructions.

### Purification of circulating CD14^+^ monocytes

EDTA whole blood from patients and healthy controls was directly subjected to peripheral blood mononuclear cell (PBMC) purification by FICOLL density gradient (LSM-1077, PAA) as previously described [31]. Monocytes were isolated by positive selection with CD14 microbeads by MACS (Miltenyi Biotec). A ≥95% purity of isolated monocytes was confirmed by flow-cytometry using antibody staining for CD14, CD16, CD3, CD4, CD8, CD19 and CD56 (BD). CXCR1 and CXCR2 staining was performed by specific antibodies (R&D); staining protocols were adapted from procedures previously established for different chemokine receptors in our laboratory [32].

### RNA isolation and gene expression analysis

RNA isolation from purified blood CD14^+^ monocytes or snap frozen whole liver tissue was conducted by pegGOLD (pegLAB). Complementary cDNA was generated with 1 µg (blood monocytes) and 2 µg RNA (liver specimen) by cDNA synthesis kit (Roche). Real-time PCR was performed with SYBR green (Invitrogen) in duplicates with β-actin as housekeeping gene [33], [34]. Primer sequences are available upon request.

### Immunohistochemistry

Frozen liver sections were stained with polyclonal rabbit anti-human MPO or monoclonal anti-human CD68 (Dako) by indirect immunoperoxidase reaction in an autostainer system (Dako). Ten visual-fields per slide were analysed at 200× magnification by a blinded investigator.

### In vitro secretion of IL-8

Monocyte-derived macrophages were obtained as described before, cultured in autologous serum and stimulated with 1 mg/ml LPS for 24 h [31].

Analyses of macrophages derived from distinct monocyte subpopulations were performed by pooling PBMC from three buffy coats of healthy blood donors. For subsequent monocyte subpopulation isolation either “monocyte-isolation-kit-II” or “CD16^+^ monocytes-isolation-kit” (Miltenyi) were used for separation of CD14^+^CD16^−^ and CD14^+^CD16^+^ monocytes respectively [4]. Isolation accuracy was confirmed by FACS analysis revealing purities >90%. Lymphocytes were obtained after removal of CD14^+^ cells as a control population. 1×10^6^ cells/petri dish were allowed to culture in RPMI medium containing 10% BSA and 1% penicillin-streptomycin (PAA) for 5 days. IL-8 concentrations in the supernatant were measured as described above.

### Statistical analysis

Statistical comparisons between two groups were performed by Mann-Whitney-U-test, multiple comparisons by ANOVA-test followed by U-test for post hoc analysis using GraphPad Prism. A p-value<0.05 was considered statistically significant. The levels of significance are indicated in the figures as followed: *p<0.05; **p<0.01; ***p<0.001. Data are depicted as bar graphs representing mean and standard error of the mean (SEM). Correlation analysis was performed by Spearman rank correlation test (SPSS).

## Results

### Serum IL-8 levels are significantly elevated in chronic liver disease, increase with stage of cirrhosis and vary between different disease etiologies

**Figure 1:**
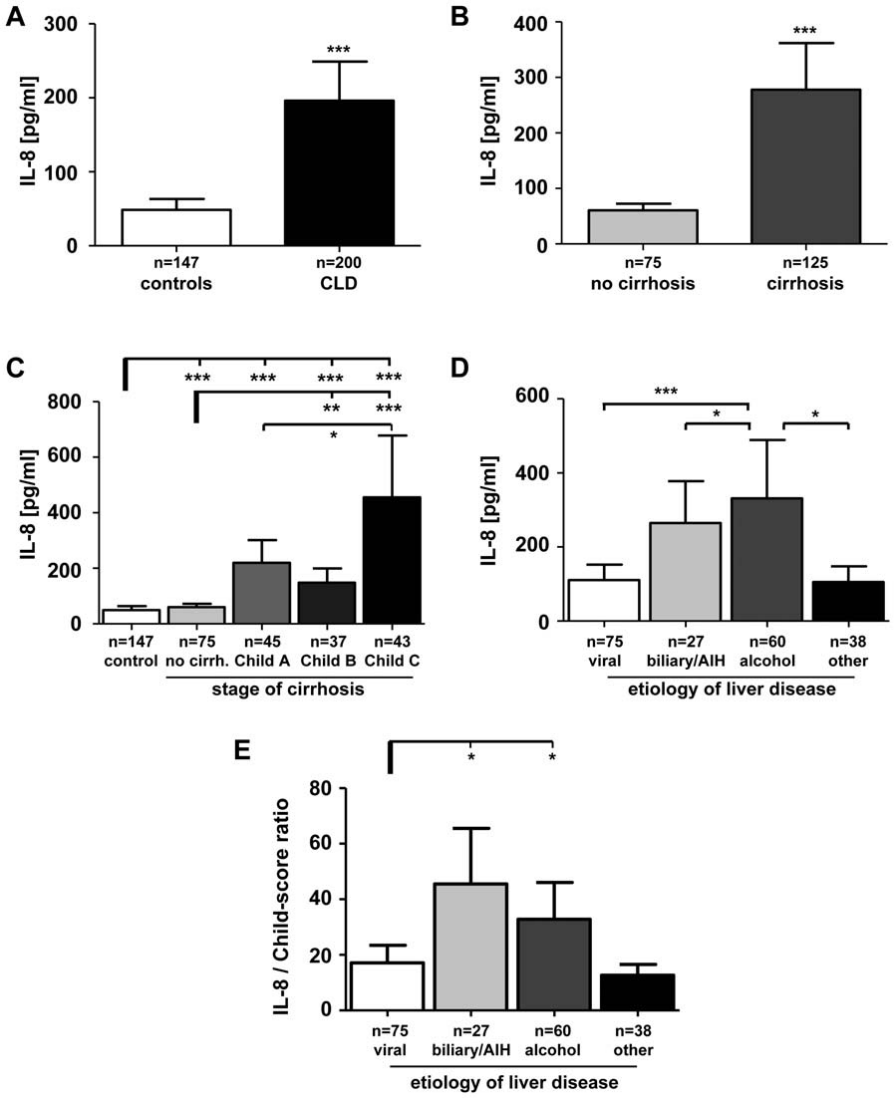
Serum IL-8 levels in healthy controls and patients with chronic liver disease. (A) Serum IL-8 levels are significantly increased in patients compared to healthy controls. (B) Cirrhotic patients show higher IL-8 serum levels than non-cirrhotics. (C) Dissecting the various stages of CLD revealed that IL-8 levels are highest in end-stage liver disease. (D) Patients with underlying alcoholic and biliary/autoimmune liver diseases showed higher IL-8 serum concentrations than viral hepatitis or other causes of CLD. (E) To normalize for the stage of cirrhosis, IL-8 serum levels were divided by the Child-Pugh score points. Elevated IL-8 in cholestatic and alcoholic liver diseases remained significant. CLD, chronic liver disease; AIH, autoimmune hepatitis.

To comprehensively address the general significance of IL-8 in the progression of chronic liver diseases (CLD), IL-8 serum levels were measured in a large cohort of patients (n = 200) in comparison to healthy volunteers (n = 147). IL-8 serum concentrations were significantly increased in CLD patients compared to controls (p<0.0001, Fig. 1A, Table 1). Of note, patients with established liver cirrhosis displayed significantly higher IL-8 levels than CLD patients without cirrhosis (p<0.0001, Fig. 1B). At all stages of liver disease (no cirrhosis, Child A, B, C), patients revealed significantly higher IL-8 concentrations than controls (Fig. 1C). Highest IL-8 concentrations were found in patients with decompensated, Child C-staged liver cirrhosis (Fig. 1C).

Interestingly, besides the clear association of IL-8 with disease progression, there were striking variations in IL-8 serum levels between the different etiologies of CLD. Patients with alcoholic, but surprisingly also with cholestatic liver diseases were found to have highest IL-8 serum concentrations (Fig. 1D), while patients with viral hepatitis (hepatitis B or C) had much lower circulating IL-8. These differences between the disease etiologies remained significant, even if IL-8 levels were adjusted to the stage of cirrhosis, which was achieved by the ratio of serum IL-8 and Child-Pugh score points (Fig. 1E). In a small subgroup of patients with PBC (n = 8) we detected by far the highest serum levels of IL-8. These findings were corroborated by correlations (Table 2) between IL-8 serum levels and parameters indicating cholestasis like total conjugated bilirubin (r = 0.394, p<0.001), gamma-glutamyl-transpeptidase (r = 0.308, p<0.001) and alkaline phosphatase (r = 0.402; p<0.001).

**Table 2:** Correlation analysis of serum IL-8 concentrations with other laboratory parameters.

	all patients (n = 200)		cirrhotic patients (n = 123)	
	r	*p*	r	*p*
***Inflammation***				
white blood cell count	0.163	*0.04*	-	*n.s.*
neutrophil count	0.155	*0.034*	-	*n.s.*
C-reactive protein	0.315	*<0.001*	0.268	*0.003*
IL-6	0.340	*<0.001*	0.350	*<0.001*
IL-10	0.220	*0.002*	0.245	*0.018*
IL-1β	0.267	*<0.001*	0.238	*0.008*
IL-4	0.243	*0.001*	-	*n.s.*
IL-2R	0.245	*0.026*	-	*n.s.*
TNFα	0.258	*<0.001*	0.342	*<0.001*
IFNy	0.241	*0.003*	0.345	*0.001*
***Chemokines***				
MCP-1 (CCL2)	0.345	*<0.001*	0.308	*<0.001*
MIP-1α (CCL3)	0.344	*<0.001*	0.334	*0.003*
MIP-1β (CCL4)	0.720	*<0.001*	0.734	*<0.001*
MIG (CXCL9)	0.340	*<0.001*	0.238	*0.008*
IP-10 (CXCL10)	0.357	*<0.001*	-	*n.s.*
***Liver function***				
MELD score	n.a.	*n.a.*	0.242	*0.008*
Child-Pugh-score	n.a.	*n.a.*	0.321	*<0.001*
albumin	−0.338	*<0.001*	−0.213	*0.020*
pseudocholinesterase	−0.337	*<0.001*	−0.235	*0.011*
prothrombin time	−0.236	*0.001*	−0.240	*0.008*
***Cholestasis***				
bilirubin (conjugated)	0.394	*<0.001*	0.368	*<0.001*
gamma-glutamyl-transferase	0.308	*<0.001*	0.220	*0.016*
alkaline phosphatase	0.402	*<0.001*	0.285	*0.002*
***Fibrosis markers***				
procollagen-III-peptide	0.349	*<0.001*	0.397	*<0.001*
hyaluronic acid	0.314	*<0.001*	-	*n.s.*

Correlations of serum interleukin-8 with other laboratory parameters were assessed by Spearman rank correlation test, and data are presented for all patients as well as for the subgroup of patients with liver cirrhosis. Only significant (p<0.05) results are shown.

IL-8 had been functionally linked to hepatic neutrophil infiltration, liver inflammation and to activation of profibrogenic, collagen-producing hepatic stellate cells [10], [27]. In fact, our correlation analysis revealed that IL-8 levels were closely linked to non-invasive markers of liver fibrosis (pro-collagen-III-peptide r = 0.349, p<0.001; hyaluronic acid r = 0.314, p<0.001) and inversely to markers of the hepatic biosynthetic capacity (prothrombin time r = −0.236, p = 0.001; pseudocholinesterase r = −0.337, p<0.001). IL-8 did not correlate with alanine aminotransferase activity (ALT), but with inflammatory cytokines and chemokines (TNFα r = 0.258, p<0.001; IL-6 r = 0.340, p<0.001; IP-10 [CXCL10] r = 0.357, p<0.001; MIG [CXCL9] r = 0.340, p<0.001; IL-4 r = 0.243, p = 0.001). Noteworthy, the counts of circulating neutrophils, that are targeted by IL-8, were also correlated with this chemokine, although the correlation was rather weak (absolute neutrophil count: r = 0.163; p = 0.004). In line with this data, we detected a significant increase of neutrophils in peripheral blood between non-cirrhotic and cirrhotic patients (p = 0.005), while neutrophil counts did not differ among the various stages of liver cirrhosis (data not shown). These findings showed that IL-8 is significantly elevated in CLD patients dependent on disease etiology and severity, indicating that increased circulating IL-8 may contribute to proinflammatory and profibrogenic cascades during the progression of chronic liver diseases.

### IL-8 and its receptor CXCR1 are up-regulated intrahepatically in chronic liver disease according to fibrosis severity and underlying pathology

**Figure 2:**
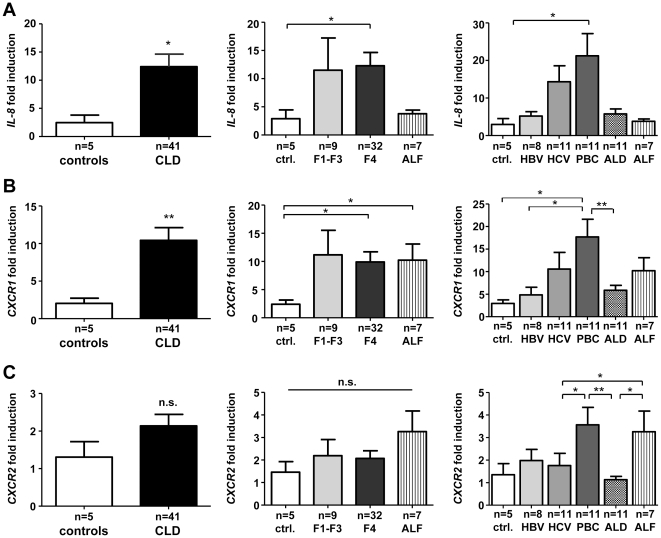
Intrahepatic IL-8 and IL-8 receptor gene expression. Gene expression levels of *IL-8* (A) and the IL-8 receptors *CXCR1* (B) and *CXCR2* (C) were assessed from liver tissue by real-time qPCR and are displayed as fold induction. Samples from CLD patients were divided according to fibrosis stage (middle graphs) and to disease etiology (right graphs). Tissue from acute liver failure (ALF) was analyzed in parallel for comparison. CLD, chronic liver disease; ctrl, control; PBC, primary biliary cirrhosis; ALD, alcoholic liver disease; ALF, acute liver failure.

Based on previous findings that several parenchymal (injured hepatocytes, biliary epithelial cells) and non-parenchymal (endothelium) liver cells as well as infiltrating immune cells are the primary sources of IL-8 in liver damage [27], [35], [36], we hypothesized that the elevated circulating IL-8 in CLD patients reflected increased hepatic IL-8 expression in liver disease. *IL-8* gene expression was significantly up-regulated in diseased liver in comparison to healthy control liver tissue by a mean of 12.4-fold (Fig. 2A). Interestingly, hepatic *IL-8* expression from CLD patients exceeded by far conditions of acute liver failure (ALF), in which IL-8-mediated neutrophil attraction has been described as an important mechanism of injury [15]. Highest *IL-8* mRNA levels were found in samples from cirrhotic livers (F4 fibrosis score), while *IL-8* induction at earlier fibrosis stages was more variable. Among the different etiologies tested, patients with primary biliary cirrhosis (PBC) exhibited highest *IL-8* mRNA expression (Fig. 2A).

We next investigated the intrahepatic expression of IL-8 receptors *CXCR1* and *CXCR2* to analyze if hepatic *IL-8* expression drives activation or infiltration of responsive cells in CLD. In full agreement with *IL-8* expression, *CXCR1* mRNA levels were significantly increased in samples from CLD patients, especially in advanced fibrosis/cirrhosis and in PBC patients (Fig. 2B). In contrast, *CXCR2* mRNA was not generally induced in CLD versus control livers, confirming prior observations [34]. However, *CXCR2* expression was increased in PBC patients (Fig. 2C). These data showed that intrahepatic expression of *IL-8* and its main receptor *CXCR1* was significantly induced in chronic liver injury, indicating that this pathway might be an important factor in driving hepatic inflammation and disease progression.

### Intrahepatic accumulation of neutrophils and macrophages in chronic liver diseases

**Figure 3:**
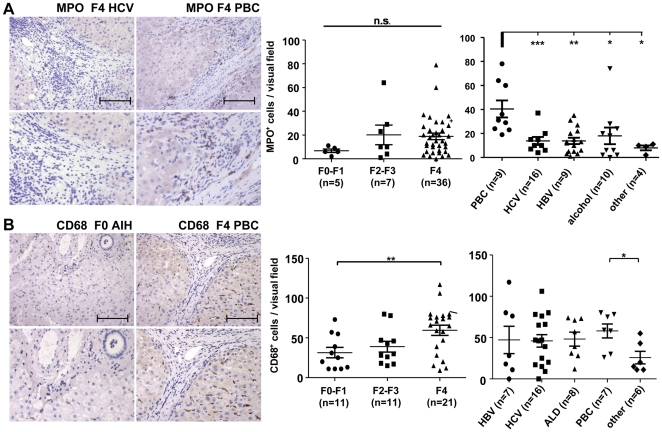
Immunohistochemical analysis of neutrophil and macrophage accumulation in chronic liver disease. (A) Myeloperoxidase (MPO) staining was performed to detect neutrophils within the liver. Upper panels depict representative images of neutrophil accumulation in PBC (left column) and HCV (right column) with F4 fibrosis at 200× magnification, lower panels magnify the periportal region of upper panels. MPO-positive neutrophils accumulate in PBC in comparison to ALD, HCV and HBV, whereas degree of fibrosis was not indicative for neutrophil count within the liver. Neutrophil cell infiltrate predominate within the cirrhotic fibres in periportal regions whereas remaining parenchyma was absent for neutrophil invasion. (B) CD68^+^ cells indicating macrophages accumulate according to severity of liver disease irrespective of underlying etiology. Images represent exemplarily distribution of CD68^+^ in F0 fibrosis (AIH) and F4 fibrosis (PBC). Scale bars represent 100 µm. AIH, autoimmune hepatitis; PBC, primary biliary cirrhosis; ALD, alcoholic liver disease.

Neutrophils have been described as a major target of IL-8 in inflammatory diseases [37]. To better understand the biological relevance of IL-8 and its receptors CXCR1 and CXCR2 in CLD we stained the liver sections for myeloperoxidase (MPO), a common marker for activated neutrophils. Although MPO^+^ neutrophils could be regularly found in livers from CLD patients by immunohistochemistry, the clear increase of *IL-8* and *CXCR1* mRNA in cirrhotic samples was not associated with increased neutrophil counts in livers with cirrhosis compared to non-cirrhotic livers (Fig. 3A). Nevertheless, there was a distinct rise of intrahepatic neutrophils in PBC, indicating that neutrophils might be specifically involved in this disease entity.

Recent studies revealed that also monocytes/macrophages may respond to IL-8, because monocytic expression of the IL-8 receptors CXCR1 and CXCR2 can be induced by several mediators like the Th2 cytokines IL-4 and IL-13 [38]. In fact, the numbers of macrophages significantly increased with progression of liver fibrosis, as evidenced by immunohistochemistry for CD68 (Fig. 3B). This finding suggested that monocytes/macrophages might be the primary responders to IL-8 in liver diseases.

### Circulating monocytes from patients with liver cirrhosis up-regulate CXCR1 expression

**Figure 4:**
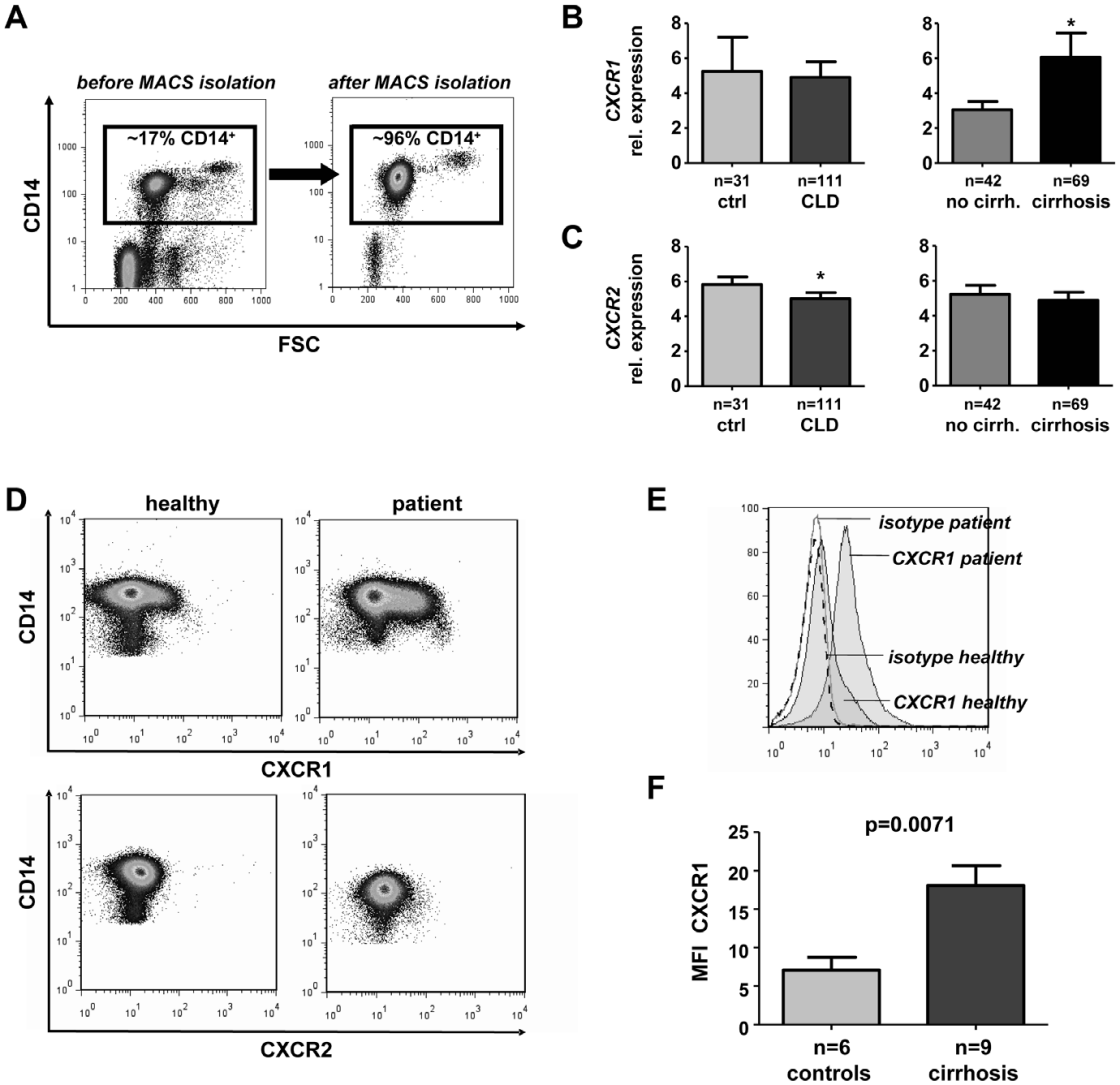
CXCR1 and CXCR2 mRNA and protein expression on circulating monocytes. (A) Monocytes were purified from whole blood by MACS with anti-CD14 antibodies, revealing purities above 95%. Representative FACS analysis before and after purification is shown. (B–C) Gene expression levels of the IL-8 receptors *CXCR1* (B) and *CXCR2* (C) were assessed from purified circulating monocytes of patients and controls and are displayed as relative expression levels. CLD, chronic liver disease. (D) CXCR1 and CXCR2 expression was also measured on a protein level by FACS. Representative plots from a healthy control (left) and patients with alcoholic Child C cirrhosis (right) are shown; pre-gating on CD14^+^ monocytes to exclude CXCR1 expression by neutrophils. (E) Representative histograms showing how mean fluorescence intensity (MFI) was assessed: isotype control staining was subtracted from CXCR1 expression on circulating CD14^+^ monocytes from healthy controls or patients. (F) Comparison of monocytic CXCR1 protein expression between healthy controls and patients with liver cirrhosis. Peripheral blood neutrophils served as a positive control for CXCR1 and CXCR2 staining (not shown).

Following the hypothesis that monocytes/macrophages are an important target of elevated IL-8 in CLD, we analyzed the expression of CXCR1 and CXCR2 by monocytes in peripheral blood. We purified circulating monocytes from healthy controls (n = 31) and CLD patients (n = 111) by CD14-labelled microbeads at a >95% purity (Fig. 4A). *CXCR1* and *CXCR2* gene expression was assessed by quantitative real-time PCR. Monocytes isolated from patients with established cirrhosis (n = 69) expressed significantly higher *CXCR1* mRNA than from non-cirrhotic patients (n = 42) (Fig. 4B). In contrast, monocytic *CXCR2* expression was moderately down-regulated in patients compared to healthy controls, independent of the presence of cirrhosis (Fig. 4C). We confirmed these findings for a subgroup of study participants on a protein level as well. In patients with liver cirrhosis, monocytic CXCR1 expression was significantly upregulated compared to healthy controls (Fig. 4D–F). CXCR2 was only barely detectable on circulating monocytes from healthy volunteers or patients (Fig. 4D). These data demonstrated that CXCR1 is upregulated on peripheral monocytes from patients with liver cirrhosis, corroborating that monocytes might be an important target of IL-8 in chronic liver diseases. Furthermore, these findings paralleled the intrahepatic expression patterns of up-regulated *CXCR1*, but not *CXCR2* (Fig. 2), in patients with liver cirrhosis. Gene expression of *CXCR3*, the receptor for CXCL9 (MIG), CXCL10 (IP-10) and CXCL11 (I-TAC), was only detected at very low levels on monocytes from healthy volunteers and even further downregulated in CLD patients (data not shown). Thus, we could exclude that macrophage infiltration was primarily driven by CXCR3 ligands, which are also upregulated during liver disease progression [39].

### Proinflammatory CD16^+^ monocyte-derived macrophages are potent producers of IL-8

**Figure 5:**
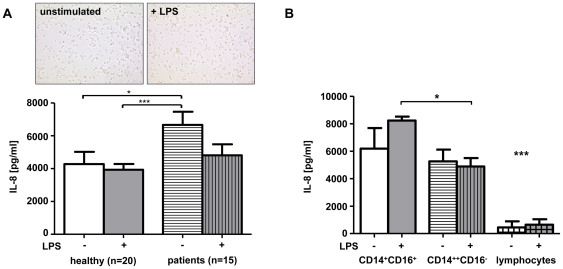
IL-8 secretion by monocyte-derived macrophages. (A) Macrophages were derived from blood monocytes of healthy donors (n = 20) and patients with Child B or C liver cirrhosis (n = 15). IL-8 secretion was measured in supernatant after 2 days of culture with autologous serum (white bars) as well as after stimulation with LPS (grey). Representative bright field microscopy pictures of cultured macrophages are displayed. (B) CD14^+^CD16^+^ monocytes, CD14^++^CD16^−^ monocytes and lymphocytes were isolated from healthy donors. IL-8 secretion was measured in supernatant after 5 days of culture with autologous serum (white bars) as well as after stimulation with LPS (grey).

To this point, our data demonstrated that IL-8 expression is induced systemically and intrahepatically in patients with CLD and that monocytes might be important responders to IL-8 during liver disease progression. However, macrophages have also been described as important IL-8 producing cells [40]. We therefore investigated whether macrophages from CLD patients may contribute to elevated IL-8 by an increased capacity to secrete IL-8. Macrophages from healthy volunteers (n = 20) and patients with liver cirrhosis (n = 15) were obtained by isolating peripheral monocytes and subsequent culture of adherent cells with autologous serum for 48 h. Macrophages from cirrhotic patients showed a significantly higher IL-8 synthesis *in vitro* (Fig. 5A). Of note, the production of other cytokines or chemokines like MCP-1, IL-10, IL-6, TNFα, MIP-1α, MIP-1β and IL-1β did not differ between patients and controls [4]. Also in contrast to other inflammatory cytokines, IL-8 production by monocyte-derived macrophages could not be further stimulated by LPS (Fig. 5A).

We had previously reported that ‘non-classical’ CD14^+^CD16^+^ monocytes, in contrast to ‘classical’ CD14^++^CD16^−^ monocytes, are critically involved in liver fibrosis progression in humans, by perpetuating a pro-inflammatory hepatic cytokine microenvironment and directly activating collagen-producing hepatic stellate cells [4]. Thus, we also compared these two major monocyte subsets for their IL-8 secretion capacity. In line with their described pro-inflammatory phenotype [31], CD14^+^CD16^+^ monocytes secreted significantly higher amounts of IL-8 *in vitro* compared to CD14^++^CD16^−^ monocytes upon LPS stimulation (Fig. 5B). Interestingly, this difference did not reach statistical significance without LPS stimulation, suggesting that endotoxemia, as often observed in advanced liver diseases [41], supports monocytic IL-8 secretion. Both monocyte subtypes were much more potent to synthesize IL-8 than lymphocytes (T-, B- and NK-cells) that were cultured as control cells (Fig. 5B). Taken together, these experiments revealed that monocytes not only respond to IL-8 in liver disease, but are also potent producers of IL-8. This indicates that IL-8, especially from ‘non-classical’ CD14^+^CD16^+^ monocytes, might contribute to amplify inflammatory signals in liver fibrosis.

## Discussion

The CXC chemokine IL-8 (CXCL8) has long been identified as a major factor of acute inflammation, acting as a potent chemoattractant and activator of neutrophils by two receptors, CXCR1 and CXCR2 [37]. High IL-8 levels in the liver and the circulation have been found in patients with acute liver injury, such as alcoholic hepatitis or ischemia-reperfusion injury [9], [10], [42]. While these conditions are clearly associated with neutrophil-mediated tissue damage [15], less data exist about the involvement of IL-8 and/or neutrophils in chronic liver injury. By comprehensively analyzing a large cohort of patients with various liver diseases, we here show that serum IL-8 levels were significantly elevated in CLD patients. IL-8 serum levels were closely associated with the progression of fibrosis/cirrhosis, as reflected by clinical scores (e.g. Child-Pugh, MELD) and laboratory tests indicating deteriorated liver function or progressed fibrosis. Thus, our data expand prior studies that were restricted to patients with HCV infection or alcoholic disease [24], [25], [26]. Moreover, our data implicate that the source of elevated circulating IL-8 in patients with chronic liver diseases is likely the injured liver, because intrahepatic *IL-8* gene expression was strongly (about 12-fold) induced in patient in comparison to control tissue.

Besides the clear association of IL-8 with disease progression, we noticed distinct differences in IL-8 serum and intrahepatic expression dependent on the underlying disease etiology. Patients with alcoholic, but surprisingly also with cholestatic liver diseases had highest IL-8 levels. Interestingly, hepatic *IL-8* expression was exceptionally high in patients with PBC. In line, IL-8 expression had been previously detected specifically in injured biliary epithelial cells in patients with cholestatic liver diseases and linked to neutrophilic infiltration around reactive bile ducts [35]. Such a specific chemoattractive role of IL-8 for neutrophil infiltration to injured bile ducts would very well explain our findings, and it would also fit to the prominent accumulation of MPO^+^ neutrophils that we noticed in livers of PBC patients.

On the other hand, the high IL-8 expression in non-biliary cirrhosis could not be attributed to a clear hepatic neutrophil accumulation in progressing fibrosis *per se*. In fact, we observed that fibrosis progression was paralleled by increasing intrahepatic *CXCR1* levels and increasing CD68^+^ hepatic macrophages. This indicated that monocytes/macrophages might be the main IL-8 responding cells in chronic liver disease patients. It has been reported previously that the Th2-cytokines IL-4 and IL-13 promote the up-regulation of CXCR1 and CXCR2 on human monocytes, thereby ‘converting’ IL-8 and related ELR^+^ chemokines, prototypic neutrophil attractants, into monocyte chemotactic agonists [38]. Interestingly, circulating monocytes from cirrhotic patients indeed had increased *CXCR1* expression, both on a gene and protein level. The enhanced monocytic CXCR1 expression could likely result from induction by Th2-cytokines, because IL-4 was found increase with progression of liver disease (not shown) and to correlate with serum IL-8 in our cohort (Table 2). These findings are in line with the current concept that advanced fibrotic disorders are generally characterized by strong Th2-biased immune responses [43].

Although our data demonstrate an association between IL-8 expression, macrophage infiltration and disease progression in patients with liver cirrhosis, the direct functional role of IL-8 in human liver disease is difficult to dissect. Functional experimental approaches with animal models are limited, because mice do not bear an IL-8 encoding gene, but only express the gene *gro*, whose gene product is the chemokine CXCL1/KC. Murine CXCL1 shows only 68% sequence identity with the human CXCL1. These differences between species are certainly a main reason why it is difficult to further dissect the role of this ELR^+^ chemokine in liver inflammation [44]. However, *in vitro* experiments characterized CXCR1^+^ macrophages as polarized towards the alternatively activated M2 macrophage phenotype [38], which is believed to act pro-fibrogenic in hepatic fibrosis [45]. Interestingly, IL-8 secreted by injured hepatocytes was shown to activate collagen-producing hepatic stellate cells *in vitro* [27]. Our study expands the profibrogenic potential of IL-8 by showing that the IL-8 signal is also amplified by ‘non-classical’, fibrosis-associated CD16^+^ monocytes/macrophages [4] and that IL-8 is in chronically inflamed liver directly associated with the number of hepatic macrophages, rather than hepatic neutrophils. Thus, our data support that IL-8 contributes to establish a profibrogenic microenvironment in chronic liver diseases and propose a novel role of IL-8 for the recruitment and activation of hepatic macrophages in human liver cirrhosis.
